# Revisiting the classical biodiversity–ecosystem functioning and stability relationships in microbial microcosms

**DOI:** 10.1093/pnasnexus/pgaf114

**Published:** 2025-04-05

**Authors:** Jiesi Lei, Jiajie Feng, Junjun Ding, Yunfeng Yang

**Affiliations:** State Key Joint Laboratory of Environment Simulation and Pollution Control, School of Environment, Tsinghua University, Beijing 100084, China; School of Biological Science and Medical Engineering, Beihang University, Beijing 100191, China; Key Laboratory of Dryland Agriculture, Ministry of Agriculture, Institute of Environment and Sustainable Development in Agriculture, Chinese Academy of Agricultural Sciences, Beijing 100081, China; State Key Joint Laboratory of Environment Simulation and Pollution Control, School of Environment, Tsinghua University, Beijing 100084, China; Institute of Environment and Ecology, Tsinghua Shenzhen International Graduate School, Tsinghua University, Shenzhen 518055, China

**Keywords:** biodiversity, ecosystem functioning, ecosystem stability, environmental stress, interspecies interaction

## Abstract

The question of how biodiversity influences ecosystem functioning and stability has been a central focus in ecological research. Yet, this question remains unresolved, primarily because of the widely divergent definitions of functioning, stability, and diversity. Consequently, forecasts of ecosystem services will remain speculative until we can establish more precise and comprehensive definitions for these concepts than previously. Here, we investigated how the maximum specific growth rate, productivity, mortality rate, and species interaction in microbial communities vary with a diversity gradient ranging from 1 to 16 species under control conditions, starvation, or saline stress. We found that diversity played a critical role in maintaining community growth and stability under control conditions, with higher diversity associated with increased maximum specific growth rate and decreased mortality rate. However, higher diversity was associated with an increased mortality rate under starvation, while diversity did not affect the mortality rate under saline stress. Diversity stabilized microbial productivity only under control conditions, defying the “diversity begets stability” hypothesis under stress. Beneficial interactions among species were prevalent in most samples, but species interaction increased mortality rates under starvation. Our findings suggest that while biodiversity is crucial for preserving ecosystem functioning and stability, the presence of multiple definitions and contextual dependence on environmental conditions argues against any general relationship between diversity and ecosystem functioning/stability. Furthermore, we provide new insights into the longstanding debate surrounding the “diversity begets stability” hypothesis and the “diversity destabilizes ecosystem” hypothesis in that diversity begets stability under control conditions but destabilizes ecosystems under severe stress.

**Significance Statement**

Understanding how biodiversity influences ecosystem functioning and stability has been a longstanding question in ecology. However, the lack of precise and comprehensive definitions for these concepts has hindered accurate predictions of ecosystem services. This study tackles this challenge by investigating the relationship between microbial community diversity and key ecological parameters under various conditions. Our results show that the popular “diversity begets stability” hypothesis is true only under control conditions, while diversity imposes contrasting effects under stress. These findings highlight the complex and context-dependent nature of the relationship between diversity and ecosystem functioning/stability, which is valuable for informing more effective strategies for preserving ecosystem functioning and resilience in the face of environmental stressors.

## Introduction

Biodiversity, the variety of life in a particular habitat or ecosystem, plays a critical role in shaping ecosystem functioning (1). However, natural ecosystems are facing various stresses imposed by climate change, landscape conversion, and other human-induced changes, all of which threaten their biodiversity, functioning, and services (2). A positive correlation between biodiversity and ecosystem functioning (BEF) has been established, particularly in studies focusing on macroscopic organisms, such as plants and animals, which play key roles in sustaining ecosystem functioning (3–5). Consequently, biodiversity preservation has been increasingly recognized as a crucial strategy for maintaining ecosystem health, with BEF research providing additional evidence to support its significance (6). However, attempts to understand the BEF relationships in the microbial world remain limited due to the complex nature of microbial communities, including high diversity, functional stability despite rapid species turnover, and prevalent high-order interactions (7). These widely observed properties make the testing of BEF in microbial communities challenging (8, 9). In a recent meta-analysis on BEF relationships across three different taxonomic groups (microbes, phytoplankton, and plants) in response to global changes, the number of experiments on bacteria, fungi, and protozoans included was only one-tenth compared with those on plants (5). Within the existing studies on bacteria, the focus has predominantly been on primary production (i.e. the maximal yield). In a study involving artificially assembled microbial communities, biodiversity did not affect microbial biomass yield but stabilized it across the treatments of warming, addition of heavy metals, and NaCl (10). Another study, which combined theoretical models and laboratory experiments with natural microbial microcosms, revealed that increased temperatures could intensify competition, potentially flattening or even reversing the BEF relationship when ecosystem functioning was represented by total biomass (11). Given the essential role of microorganisms in driving biogeochemical cycles (12), it is imperative to investigate multiple key indices of microbial physiology that are closely linked with ecosystem functioning (13). While primary production is a well-recognized ecosystem function, the growth rate and mortality of microbial communities are also crucial for understanding ecosystem dynamics. Microbial growth rate influences nutrient uptake and conversion, thereby playing a vital role in nutrient cycling and energy flow (14–17). Similarly, microbial mortality contributes to biomass turnover and nutrient recycling, which are fundamental processes in biogeochemical cycles (18). These population-level attributes extend to a community of microbes, encompassing different species and their interactions, thus providing insights into BEF relationships.

Several hypotheses have been proposed to explain both BEF and biodiversity–ecosystem stability (BES) relationships, including the diversity–stability hypothesis (19), rivet hypothesis (20), redundancy hypothesis (21), and insurance hypothesis (10, 22). These frameworks share a common foundation: biodiversity can enhance ecosystem stability and resilience, though the mechanisms vary. While originally developed for macroorganisms, these hypotheses may also be relevant in microbial communities, where high functional redundancy, rapid species turnover, and metabolic plasticity influence stability–functioning relationships. Empirically, a conspicuous BES relationship implies that a decline in diversity may lead to the destabilization of ecosystems. However, the prevalent belief that diversity begets stability was dispelled by a theoretical prediction that more diverse ecological communities were more unstable if species interactions were intense (23). Resolving this paradox is a key to understanding whether microbial diversity enhances or weakens stability under different environmental conditions.

**Fig. 1.**
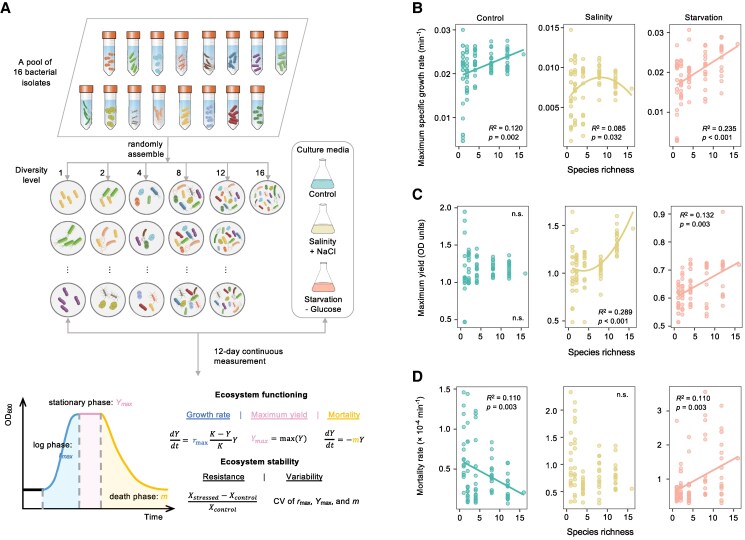
Effects of treatment and biodiversity on community growth rate, yield, and mortality rates. A) Experimental schematic for testing the BEF and BES relationships in the face of environmental stress. Communities were assembled by combining a defined number of organisms at equal ratios to form a diversity gradient of 1, 2, 4, 8, 12, and 16 species. Environmental stress application involved exposing the assembled bacterial communities to one of three conditions—control, saline, or starvation stress—after a growth period of 12 h at ambient temperature. Growth curve analysis was conducted using a BioScreen C Microbiological Growth Analyzer to continuously monitor community biomass over a 12-day period. The growth curves were deconstructed into distinct phases, and mathematical models were employed to extract parameters, such as maximum growth rates during logistic growth, peak biomass achieved by the communities, and mortality rates during the decline. Stability metrics, including ecosystem resistance to environmental stress and the CV, were derived to evaluate the resistance and variability of community functioning under stress (see Methods). B–D) Relationship between taxonomic diversity measured by species richness and B) maximum specific growth rate, C) maximum yield (OD_600_ values), and D) mortality rates under different treatments. Biodiversity is measured as species richness. The mean values of four replicates are shown in the figure. n.s. indicates that the regression is not significant at the 0.05 threshold, and the regression line is omitted.

Here, we investigated the BEF and BES relationships in bacterial communities subjected to control conditions or two distinct environmental stresses, i.e. saline stress and starvation (Fig. 1A). Our initial focus was to observe the overall BEF and BES patterns by minimizing the emphasis on the identity and composition of bacterial species. To achieve it, we randomly assembled artificial microbial communities from a pool of 16 proteobacterial species, one of the most ecologically dominant and metabolically diverse bacterial phyla in natural environments (24, 25), thus asking a simplified question primarily about diversity with a gradient of 1, 2, 4, 8, 12, and 16 species (Tables S1 and S2). Subsequently, we investigated the mechanisms by considering the roles of species composition, tolerance, and ecological strategies indicated by rRNA operon copy number. We expanded the scope of ecosystem functioning beyond the commonly measured maximum yield by incorporating the maximum specific growth rate (i.e. the highest rate of increase in biomass of a cell population per unit of biomass concentration) and the mortality rate reflecting the community’s vitality and structure, which has not been considered in any previous BEF study, to our knowledge. We evaluated ecosystem stability by quantifying ecosystem resistance to environmental stress and variability assessed as the coefficient of variation (CV) of functioning. We calculated biodiversity using both species richness and phylogenetic diversity (PD), as the latter integrates evolutionary distances among species, which can serve as a proxy for ecological differentiation and functional complementarity. Our selected Proteobacteria spanned a relatively broad phylogenetic range (PD: 0–0.8) but were primarily concentrated in γ-Proteobacteria, enabling us to examine both phylogenetically diverse and closely related communities. By measuring community growth every 30 min for 12 consecutive days, we collected experimental data to infer the maximum specific growth rate, maximum yield, mortality rate, and species interactions. Species interactions in our experiment refer to the effects that species within a community have on each other's growth and survival. We quantified these interactions by comparing the observed community performance to the expected performance based on monoculture measurements and the proportion of the species within the community of the inoculum. Positive interactions occur when the community performs better than expected, indicating synergistic effects, while negative interactions occur when the community performs worse than expected, indicating competitive or inhibitory effects. As it is highly uncertain how different environmental stressors might interact with biodiversity to affect ecosystem functioning and stability, we hypothesized that the relationship between biodiversity and ecosystem properties would not be uniform but would instead hinge on the specific type of stressor imposed—starvation or saline stress.

## Results and discussion

### The BEF relationships

We measured the maximum specific growth rate by identifying the logarithmic phase of growth curves from which it is derived, representing the rapid increase in biomass per unit of biomass concentration and serving as a proxy for an ecosystem's functional efficiency. The maximum specific growth rate was strongly affected by taxonomic diversity calculated by species richness and PD calculated by Faith's PD (*P* < 0.001; Table 1), with directions and magnitudes dependent on the treatment type (*P* < 0.005; Table 1). To depict the BEF relationships, we carried out both quadratic and linear regression analyses between diversity and maximum specific growth rates and retained the model with the better fit (Fig. 1B). Positive diversity-maximum specific growth rate correlations were observed for control samples (*P* = 0.002). In contrast, maximum specific growth rates exhibited unimodal, hump-shaped relationships with taxonomic diversity under saline stress (*P* = 0.032), with a peak maximum specific growth rate at the mid-diversity level, suggesting that the diversity-maximum specific growth relationship is context dependent. These different patterns could arise from general BEF mechanisms. Specifically, selection and complementary effects could help maintain community growth beyond the peak of the taxonomic diversity hump (22); however, they could be offset by competitive, negative interactions that are prevalent in communities with high diversity (26, 27), resulting in diverse BEF relationships. When subjected to starvation, maximum specific growth rates increased linearly with both taxonomic and PDs (*P* < 0.001) and at a faster pace than under control (slope = 6.46 × 10^−4^ under starvation vs. 3.81 × 10^−4^ under control for species richness, Fig. 1B; slope = 11 × 10^−3^ under starvation vs. 5.58 × 10^−3^ under control for faith's PD; Fig. S1A). Therefore, high diversity led to a more pronounced increase in maximum specific growth rate in nutrient-scarce environments, which might result from beneficial species interactions such as mutual metabolite exchange to alleviate resource limitation (28, 29).

**Table 1.** Effects of treatment and biodiversity on microbial community growth, yield, and mortality rate.

Effect	Maximum specific growth rate			Maximum yield			Mortality rate		
	df	*F*	*P*-value	df	*F*	*P*-value	df	*F*	*P*-value
Species richness	1	37.855	**<0**.**001**	1	19.531	**<0**.**001**	1	0.463	0.497
Type of treatment	2	266.627	**<0**.**001**	2	234.582	**<0**.**001**	2	18.377	**<0**.**001**
Species richness × treatment	2	5.514	**0**.**005**	2	7.075	**0**.**001**	2	13.691	**<0**.**001**
Phylogenetic diversity	1	28.023	**<0**.**001**	1	15.933	**0**.**023**	1	0.101	0.751
Type of treatment	2	400.214	**<0**.**001**	2	331.908	**<0**.**001**	2	20.864	**<0**.**001**
Mean pairwise distance × treatment	2	8.213	**<0**.**001**	2	8.865	**<0**.**001**	2	8.452	**<0**.**001**

The types of treatments include control, saline stress (i.e. additional NaCl), and starvation (i.e. reduced nutrients). × denotes an interactive effect. Significant *P*-values (*P* < 0.050) are marked in bold.

The diversity-maximum yield relationships varied between the control and stressed samples (Figs. 1C and S1B). In control samples, there was no significant correlation between maximum yield and diversity (*P* > 0.050), suggesting that the commonly observed positive diversity–productivity relationship in plant ecology, particularly when diversity is low (30), may not be applied in microbial ecology. In contrast, maximum yield under saline stress was high when microbial diversity was high (*P* < 0.001). This result was a notable deviation from the previous observation, wherein fewer species and milder stress were tested, that maximum yield exhibited a hump-shaped relationship with microbial diversity under both control and stressful conditions (10). However, our result is partly in line with the yield–acquisition–stress (Y-A-S) framework (31), which classified microbial life-history strategies into three main categories: high yield (Y), resource acquisition (A), and stress tolerance (S) across the gradients of resource availability and stress. The Y-A-S framework predicts that microorganisms under saline stress would sacrifice the growth yield to maintain cellular integrity and osmotic balance through the production of osmolytes such as trehalose, glycine betaine, or other extracellular polymeric substances to protect cells from desiccation (32, 33). This leads to increased resource demand and competition as diversity increases, which may explain the initial decrease in community growth yield with diversity. However, the positive correlation observed between diversity and community growth yield at higher diversity levels (Fig. 1C) suggests that species interactions might play a role.

**Fig. 2.**
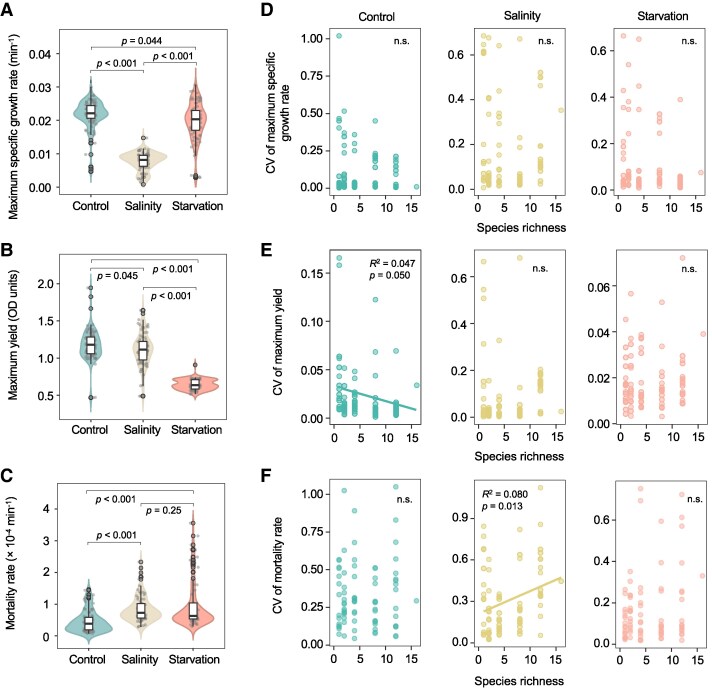
Effect of biodiversity on the CV of ecosystem functioning metrics under control and treatments. A–C) Community stability was quantified by ecosystem resistance to environmental stress, measured as the stressor-induced changes in ecosystem functioning metrics: A) maximum specific growth rate, B) maximum yield (OD_600_ values), and C) mortality rates. D–F) Alternatively, community stability was quantified by the CV of these ecosystem functioning metrics: D) community maximum specific growth rates, E) maximum yield (OD_600_ values), and F) mortality rates under control and treatments. n.s. indicates that the regression is not significant at the 0.050 threshold, and the regression line is omitted.

We calculated the mortality rate by fitting exponential models to the death phase of the growth curves, reflecting the rate at which biomass is lost and representing systemic vulnerability to environmental stress. The mortality rate was strongly affected by treatment type (*P* < 0.001; Table 1) and by the interaction between treatment type and both taxonomic and PDs (ANOVA, *P* < 0.001). The mortality rate was increased by an average of 86.2% under saline stress and 110.4% under starvation (*P* < 0.001; Fig. 2C), verifying the detrimental effects of environmental stressors on microorganisms. Although starvation reduced the maximum biomass more than saline stress did (Fig. 2B), there was no significant difference in the mortality rates between saline stress and starvation (*P* = 0.250; Fig. 2C), suggesting that the mortality rate is influenced by factors other than biomass reduction.

Higher taxonomic diversity was associated with a reduction in mortality rates in control samples (*P* = 0.003; Fig. 1D), suggesting that high diversity empowered community sustainability. This relationship may be due to the complementary functional and metabolic capacities of individual members of the community, which may contribute to their overall performance (34). However, the mortality rates under saline stress remained similar across the diversity gradient, while the mortality rates under starvation increased with higher taxonomic and PDs (*P* < 0.001; Figs. 1D and S1C).

### The BES relationships

**Fig. 3.**
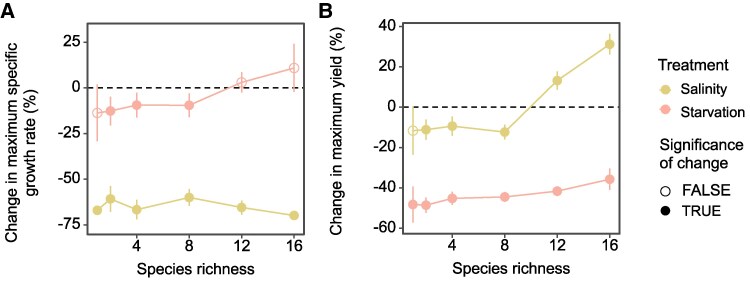
Effect of biodiversity on community stability. Community stability was quantified by ecosystem resistance to environmental stress, that is, the stressor-induced changes in A) maximum specific growth rate and B) maximum yield (OD_600_ values). The relative change is presented as the mean (circle) with a 95% CI (line), and significant changes under stressed conditions compared with the control are represented by filled circles.

The definition of stability is multifaceted. We first quantified stability in terms of resistance, by measuring the differences in maximum specific growth rate, maximum yield, and mortality rates between control and stressful conditions. Compared with control samples (i.e. control condition), both saline stress and starvation reduced the maximum specific growth rates of microbial communities (Fig. 2A). The average reduction was 64.1% (*P* < 0.001) under saline stress, much higher than that of starvation (7.7%, *P* = 0.044) since the higher resource utilization efficiency induced by starvation may have potentially offset food scarcity (35). Both stressors also reduced maximum yield (Fig. 2B), consistent with the Y-A-S framework that predicts greater energy investments in maintenance activities or resource uptake in harsh environments at the expense of growth yield (31). Starvation reduced maximum yield by 45.4% (*P* < 0.001), much higher than that of saline stress (−5.8%, *P* = 0.045). Under starvation, compared with control conditions, the most notable reductions in maximum specific growth rate and maximum yield were observed at low diversity levels (Fig. 3A and B). However, these reductions became less pronounced at higher diversity, suggesting that diversity enhanced ecosystem resistance. For saline stress, the reduction in maximum yield decreased with increasing diversity (maximum yields under saline stress were even significantly [*P* < 0.050] higher than control at high diversity levels; Fig. 3B), while that of maximum specific growth rate remained largely unchanged (Fig. 3A).

We then calculated the CVs of ecosystem functioning metrics to quantify the relative variability across biological replicates of ecosystem functioning around the mean value. In line with previous studies (10, 36), the CV of maximum yield across biological replicates decreased linearly with diversity in control samples (*P* = 0.050; Fig. 2E), suggesting that higher biodiversity reduced biomass variability, which supports the notion of “diversity begets stability” (19). Under saline stress, a positive correlation between CV of maximum yield and PD was observed (*P* = 0.015; Fig. S2B), which rejected the previous observation that biodiversity acted as insurance of bacterial maximum yield (10). However, no such correlation was found under starvation or between CV of maximum specific growth rate and diversity (Figs. 2D and S2A). The CV of community mortality rates remained largely constant across the diversity gradient under controlled and starvation conditions (Fig. 2F), aligning with more recent findings (37). In contrast, the CV of mortality rates increased linearly with biodiversity when subjected to saline stress (*P* = 0.013). Therefore, more diverse communities could exhibit higher variability in their response to saline stress.

### Stress-tolerant versus stress-sensitive species

We define species whose maximum yield in monoculture was significantly (*P* < 0.050) decreased by saline stress or starvation as stress-sensitive species, and otherwise stress-tolerant species. We identified eight out of 16 species as salt tolerant and two out of 16 species as starvation-tolerant (Fig. S3), with *Cupriavidus necator* H16 and *Asticcacaulis taihuensis* T3-B7 displaying resistance to both stresses. Both *C. necator* H16 and *A. taihuensis* T3-B7 had lower copy numbers of ribosomal RNA operons (*rrn*) than other species (Table S1), suggesting that they were slow in growth but more efficient in substrate use (38). As more than half of the constituent species in our study were salt tolerant, the high prevalence of salt-tolerant species may have masked the effect of biodiversity on community resistance with regard to maximum specific growth rate. The increase in the number of tolerant species with diversity under saline stress (*r* = 0.936, *P* < 0.001; Fig. S4) may explain the observed decrease in community mortality with diversity. However, the number of tolerant species was fewer under starvation conditions, suggesting that diversity may have a limited ability to mitigate the effects of this stressor. Artificial communities dominated by those salt-tolerant species (i.e. over half of the constituent species were salt tolerant) also showed a U-shaped correlation between diversity and maximum yield under saline stress (Fig. S5), suggesting that the presence of salt-tolerant species inhibited the diversity-maximum yield relationship when diversity was low but enhanced it at higher diversity.

We used linear mixed models to unveil the contributions of those tolerant species to maximum specific growth rates, maximum yield, and mortality rates subjected to different stressors, controlling the factor of diversity (both species richness and Faith's PD) that was generally positively associated with the number of tolerant species due to “sampling effects” (39). Under saline stress, the number of tolerant species did not contribute to the maximum specific growth rate (*χ*^2^ = 0.078, *P* = 0.780; Table 2); therefore, sensitive species would be equally important as tolerant species for the maximum specific growth rate. However, the growth of sensitive species is unlikely without the help from tolerant species because the maximum specific growth rate of sensitive species monoculture was decreased by saline stress (Fig. S3). Therefore, species interaction would be essential for sensitive species to grow.

**Table 2.** Effects of biodiversity and the presence of tolerant species on microbial community growth, yield, and mortality rate.

	Maximum specific growth rate			Maximum yield			Mortality rate		
	*β*	*χ* ^2^	*P*-value	*β*	*χ* ^2^	*P*-value	*β*	*χ* ^2^	*P*-value
Species richness as a random effect									
Saline stress	3.59 × 10^−5^	0.078	0.780	0.027	5.319	**0**.**021**	−0.009	0.077	0.855
Starvation	−0.001	5.335	**0**.**021**	−0.013	4.241	**0**.**039**	−0.287	14.154	**<0**.**001**
Phylogenetic diversity as a random effect									
Saline stress	4.27 × 10^−5^	0.105	0.746	0.042	19.895	**<0**.**001**	−0.054	2.267	0.132
Starvation	0.001	3.910	**0**.**048**	0.015	1.761	0.185	0.080	0.400	0.527

Species whose maximum yield in monoculture was significantly (*P* < 0.050) decreased by saline stress or starvation are defined as sensitive species, while species that were unaffected are considered tolerant. × denotes an interactive effect. Significant *P*-values (*P* < 0.050) are marked in bold.

Under saline stress, the number of tolerant species was positively associated with maximum yield (*β* = 0.027, *χ*^2^ = 5.319, *P* = 0.021 when species number was controlled for; *β* = 0.042, *χ*^2^ = 19.895, *P* < 0.001 when PD was controlled for; Table 2). This indicates that, if biodiversity is equal, the tolerance of constituent species to saline stress plays an important role in achieving the maximum yield. In sharp contrast, under starvation, the number of tolerant species reduced maximum specific growth rates (*β* = −0.001, *χ*^2^ = 5.335, *P* = 0.021) and maximum yield (*β* = −0.013, *χ*^2^ = 4.241, *P* = 0.039). Microbes adapted to oligotrophic environments often outcompete copiotrophs, which thrive in nutrient-rich conditions but tend to have lower resource-use efficiency. This adaptation allows oligotrophs to achieve more efficient nutrient uptake and storage (40), which may further exacerbate the adverse effects of nutrient scarcity on the growth of other bacteria. However, while tolerant species had a detrimental effect on community growth under starvation, they made a significant contribution to community stability, as mortality rates decreased with an increasing number of tolerant species (*β* = −0.287, *χ*^2^ = 14.154, *P* < 0.001; Table 2).

### Strength and direction of species interaction

**Fig. 4.**
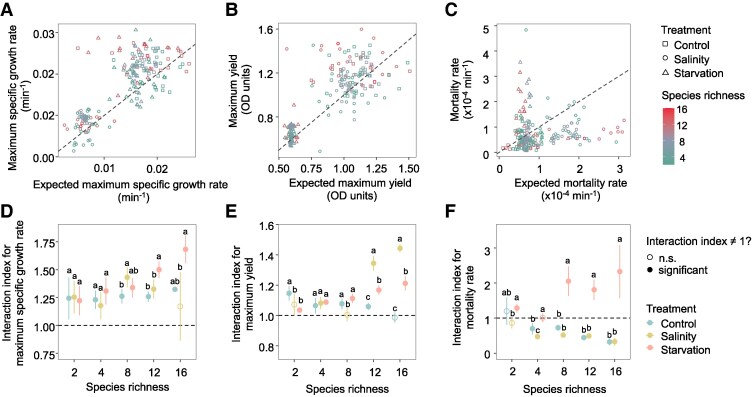
Strength and direction of species interaction. A) The observed maximum specific growth rates for communities with two or more species plotted against the weighted sum of the monoculture growth rates of constituent species. B) The observed maximum yields for communities with two or more species plotted against the weighted sum of the monoculture yields of constituent species. C) The observed mortality rates for communities with two or more species plotted against the weighted sum of the monoculture mortality rates of constituent species. Square = control; circle = salinity; triangle = starvation. The dashed line shows the 1:1 relationship. D) Interaction index for maximum specific growth rate, calculated as the ratio of observed to expected additive maximum specific growth rates, is shown across different richness levels. E) Interaction index for community yield, calculated as the ratio of observed to expected additive maximum yields, is shown across different richness levels. F) Interaction index for mortality rate, calculated as the ratio of observed to expected mortality rates, is shown across different richness levels. An interaction index of 1 would be additive, that is, no interaction. The interaction index is summarized as mean (circle) and its 95% CIs (line) with filled circles denoting significant interactions whose 95% CIs do not overlap 1. Differences in mean interaction index values under control, saline stress, and starvation at each richness level were tested with ANOVA followed by Duncan's test, and are marked with different letters.

We quantified species interaction through the comparison of ecosystem functions (i.e. maximum specific growth rates, maximum yield, and mortality rate) in mixed species communities relative to those in monocultures. For community maximum specific growth and maximum yield, positive interactions are inferred when species mixtures exhibit higher functions than predicted by the sum of their monocultures weighted by abundance (synergistic effect), while negative interactions denote a lower-than-additive performance (41). For these two metrics, positive interactions occurred more frequently than negative ones (Fig. 4A and B). On average, the maximum specific growth rates of species mixtures were 24.9 ± 2.7% higher than the additive values under controlled conditions, similar to the observed values under saline stress (24.9 ± 1.0%), but lower than that under starvation (34.7 ± 2.7%; *P* < 0.050; Fig. 4A). Likewise, the maximum yield of species mixtures was on average 8.5 ± 1.2% higher than the additive values under control, similar to the observed values under saline stress (10.2 ± 1.6%), but lower than that under starvation (13.4 ± 0.7%; *P* < 0.050; Fig. 4B). For mortality rates, a high value indicates weakened ecosystem functioning; thus, positive interactions occur when observed values are lower than the abundance-weighted additive values, indicating a synergistic effect that reduces mortality rates. Conversely, negative interactions increase mortality rates. Positive interactions for mortality rates were more prevalent under controlled conditions and saline stress, as evidenced by 24.1 ± 5.6 and 41.9 ± 2.7% reductions in mortality rates compared with the additive values, respectively (Fig. 4C). In contrast, the observed mortality rates were, on average, 55.3 ± 7.3% higher than that of additive values under starvation, suggesting that species interaction accelerated microbial death when food was scarce.

We observed significant differences in the strength and direction of interactions (*P* < 0.050, one-way ANOVA followed by Duncan's multiple range test; Fig. 4D–F). Starvation enhanced positive interactions in 12 and 16 species communities for maximum specific growth rates, which were greater than those under control or saline stress (*P* < 0.050, ANOVA followed by Duncan test; Fig. 4D). Conversely, the strength of positive interactions for maximum yield was strong under saline stress, considerably higher than those under control and starvation (*P* < 0.050; Fig. 4E). Negative interactions for community mortality rates were evident in communities with more than four species under starvation, while significant, positive interactions were found in samples under control or saline stress in these communities (Fig. 4F). The prevalence of positive interactions detected in stressful environments lent partial support to the refined stress gradient hypothesis or “hunger games” hypothesis, which proposes that facilitation should be more common in stressful environments (26). Our findings on growth rates and yields align with observations from other ecosystems (42, 43), where resource scarcity enhances facilitation among community members. This suggests a general principle of ecosystem adaptation to stress, in which facilitative interactions become more pronounced when resources are limited. However, the strength of interactions under salt stress, measured by the maximum specific growth rate, was comparable with the strength of interactions observed in the control sample (Fig. 4D), which belied this notion.

### Limitations and outlook

It is important to note that the results of our study, as well as similar studies, have limitations in their generalizability to natural conditions. Although we included a broad phylogenetic range of proteobacterial species (PD: 0–0.8), the diversity of our system was lower than natural communities by several orders, as soils exhibit a bacterial PD on the order of 10^1^–10^2^ (44, 45), and the human gut microbiome has a PD on the order of 10^1^ (46). As a result, we observed weaker correlations between ecosystem functioning and PD compared with species richness in our experiments. In addition, our observations were based on the short-term microbial response to an environmental stressor. It is possible that competitive relationships among species may change with long-term or recurrent exposures. As natural communities are complex ecosystems with myriads of biotic and abiotic interactions, it remains unclear whether the lessons learned from artificial communities can be applied to natural microbial communities or even ecological communities across different scales. Moreover, our experimental design was to randomly assemble artificial microbial communities to minimize the emphasis on the identity and composition of bacterial species. As a result, some species are overrepresented in the experiment, which can have consequences for the response variables. In future studies, it would be beneficial to examine the specific roles of overrepresented species in driving the observed patterns, which could provide deeper insights into the mechanisms underlying BEF and BES relationships. Lastly, we assessed growth and mortality rates based on total community biomass (OD_600_), which does not resolve species-specific contributions. The reason is that the total volume of each culture is merely 1,280 μL, which is insufficient for time-series sampling. In addition, sampling will impose substantial disturbances to bacterial cultures.

Our study confirms the critical role of biodiversity in sustaining ecosystem functioning and stability under controlled conditions (22). However, this relationship may not hold under stressful environments, suggesting that ecosystem stability cannot be universally characterized. The context dependency of the BEF and BES relationship may be attributed to changes in the species interactions, which can have both positive and negative effects on community functioning and stability (41). In addition, high levels of biodiversity may strengthen negative species interaction (competition) but may also give more opportunities for positive species interaction (mutualistic metabolic exchanges). Starvation acts as a direct constraint on resource availability, limiting the energy and nutrients necessary for growth. In contrast, salinity imposes an environmental stress that affects the community's physiology and survival directly. Both stressors, therefore, impose selective pressures that mold life-history traits, including growth and death rates, by prioritizing energy conservation and efficiency. By considering these first principles, we can begin to comprehend how changes in the energy budget under different stress conditions can influence the balance of positive and negative species interactions. By framing our results within these foundational ecological concepts, the implications extend beyond microbial microcosms to broader ecosystem contexts.

Overall, our findings provide valuable insights into the context dependency of BEF and BES relationships. Future experiments involving a larger species pool and long-term or recurrent exposures to environmental stresses will be needed to generalize the observed patterns to natural conditions. In addition, our empirical findings lay the groundwork for theoretical models, such as consumer-resource and Lotka–Volterra models, to test assumptions related to species interactions and stress responses. It is likely to incorporate a diversity of resource types, integrate empirically derived species traits and interaction parameters, and model facilitative interactions to capture cooperative behaviors influencing community stability under stress. This dual approach of empirical and theoretical frameworks will enrich our exploration of BEF and BES relationships.

## Materials and methods

### Bacterial species

Bacterial strains used in this study were obtained from the microbial culture collections of the Institute of Microbiology, Chinese Academy of Sciences (IMCAS), and the School of Life Sciences, Tsinghua University, Beijing, China (Table S1). A total of 16 bacterial species from different phylogenetic groups within the phylum Proteobacteria were selected to encompass a relatively broad phylogenetic range while preserving a certain level of genetic conservation. Specifically, two species were selected from each of the α-Proteobacteria (*A. taihuensis* and *Novosphingobium taihuense*) and β-Proteobacteria (*C. necator* H16 and *Undibacterium terreum*), and 12 species were selected from the γ-Proteobacteria group (*Aeromonas hydrophila* 4AK4, *Escherichia coli* DH5α, *E. coli* MG1655, *Stutzerimonas stutzeri* 1317, *Pseudomonas putida* KT2442, *Proteus vulgaris*, *Aliiglaciecola lipolytica* E3, *Salmonella Braenderup* H9812, *Klebsiella pneumoniae*, *Vibrio cholerae*, *Acinetobacter baumannii*, and *Salmonella typhimurium* ATCC 14028). The strains were selected based on their cultivability and compatibility in the microcosom setup. The ribosomal RNA Database (rrnDB) (47) was searched to estimate the ribosomal RNA operon (*rrn*) copy number for each species, starting from its lowest taxonomic rank. In cases where multiple matches were available for a given strain, the median *rrn* copy number was used from all available records (Table S1). The 16S rRNA gene sequences for these 16 bacteria and three outgroup species (*Deinococcus misasensis*, *Halobacterium rubrum*, and *Thermomicrobium carboxidum*) were aligned using ClustalW to build a Maximum Likelihood phylogenetic tree using MEGA 11 (48) (Fig. S6). Using the generated tree, the PD was calculated as the sum of tree branch lengths connecting the present species together (49), and the mean pairwise distance was calculated as the average phylogenetic distance connecting all species present in a community (50), using the R package *picante*.

### Microcosm assembly and growth assays

Bacterial cultures (700 μL) were cryopreserved in glycerol stocks (30%, 700 μL) at −80 °C and grown in a rich medium (10 g L^−1^ tryptone, 10 g L^−1^ sodium chloride, and 5 g L^−1^ yeast extract). Following overnight incubation at ambient temperature, the bacterial species were assembled into various combinations with a gradient of diversity ranging from 1 to 16 (1, 2, 4, 8, 12, and 16; Table S2) through random samplings from a pool of 16 species. Following the methods in Awashi et al. (10), the combination of bacterial species was independently, randomly generated sixteen times at richness levels of 2, 4, 8, and 12, resulting in 64 combinations. Therefore, the total number of combinations was 81, after taking into account 16 monocultures and 1 combination at the richness level of 16 (SI Appendix, Table S2). Each combination was replicated four times, generating a total of 324 microcosms for each treatment. For each assemblage, an equal amount of inoculum of each species was taken to form microcosms with a final volume of 1,280 μL. For instance, to create a microcosm with a single species, 1,280 μL of the corresponding inoculum was taken, whereas for a four-species assemblage, 320 μL of each constituent strain's inoculum was taken and combined. The optical density (OD_600_) of each inoculum was kept consistent, ensuring equal cell density across all microcosms. As a result, the final cell density in each microcosm was the same.

Environmental stresses (i.e. saline stress and starvation) were imposed on bacterial cultures 12 h after inoculation at 25 °C. To achieve this, we used a minimum medium comprised of: 2.0 g L^−1^ (NH_4_)_2_SO_4_, 0.2 g L^−1^ MgSO_4_, 9.65 g L^−1^ Na_2_HPO_4_·12H_2_O, 1.5 g L^−1^ KH_2_PO_4_, 10 mL L^−1^ trace element solution I, and 1 mL L^−1^ trace element solution II. The trace element solution I consisted of 5 g L^−1^ Fe(III)-NH_4_-citrate, 2 g L^−1^ CaCl_2_, and 1 M HCl. The trace element solution II contained: 100 g L^−1^ ZnSO_4_·7H_2_O, 30 g L^−1^ MnCl_2_·4H_2_O, 300 g L^−1^ H_3_BO_3_, 200 g L^−1^ CoCl_2_·6H_2_O, 10 g L^−1^ CuSO_4_·5H_2_O, 20 g L^−1^ NiCl_2_·6H_2_O, 30 g L^−1^ NaMoO_4_·2H_2_O, and 0.5 M HCl. For the control group, 20 g L^−1^ glucose was added as the sole carbon source to the minimum medium, while for the starvation group, the amount of glucose added was 0.5 g L^−1^. For the saline stress group, we maintained 20 g L^−1^ glucose in the minimum medium but added NaCl to a final concentration of 0.7 M (equivalent to 40 g L^−1^). The culture media were added to 100-well Honeycomb sterilized plate readers (297 μL per well). Aliquots from microcosms (3 μL each) were then transferred into the different media and incubated at 25 ± 1 °C for 12 days in a BioScreen C Microbiological Growth Analyser (Labsystems, Helsinki, Finland) with continuous shaking. The OD was automatically recorded every 30 min using a 600 nm filter for 12 days. The 12-day duration for the microcosm experiment was chosen based on our preliminary experiments, which showed that 12 days is enough time to capture the full growth patterns of microbial communities. The data were recorded and processed using the Easy BioScreen Experiment (EZ Experiment) software provided by the manufacturer and then exported to Microsoft Excel for further analysis.

### Statistical analyses

To analyze the effects of biodiversity on ecosystem functioning, we developed a MATLAB script to reconstruct growth curves and calculate the growth rate, mortality rate, and maximum yield for each microcosm. The script (i) detects outlier points and smooths the growth curves with a moving average. Outliers were defined as data points that deviated >5% from the mean of the preceding and following points within a specified window size. These outliers were replaced with the average of the means of the preceding and following windows to ensure accurate curve fitting; (ii) calculates the gradient of the growth curves and segments the logarithmic growth phase, steady phase, and death phase based on predetermined thresholds; (iii) fits the logistic model to the logarithmic growth phase to calculate the maximum specific growth rate, and fits the exponential model to the death phase to calculate the mortality rate; and (iv) records the maximum OD values and the mean of three consecutive values around the max as maximum yield. The three consecutive values include the maximum value and the data points immediately before and after it, providing a more robust estimate of the peak biomass. Subsequent statistical analyses were carried out using R software 4.0.3 with packages *lme4* and *agricolae*, unless otherwise indicated. To determine the tolerance of species to environmental stressors, we calculated the percentage change in the maximal yield of monocultures in the absence (control) and presence of stressors using data from the replicates of monocultures. The significance of the percentage changes was determined using Student's t test. Species whose biomass in monoculture was not reduced in the presence of a stressor were considered tolerant. To quantify community stability, we employed two measures: ecosystem resistance to environmental stress and the CV. Ecosystem resistance to environmental stress was determined by calculating the percentage change in the community's maximum specific growth rate or yield under stress conditions compared with that under controlled conditions. The statistical significance of these relative changes was assessed using Student's t test. The CV was calculated based on biological replicates within each treatment to assess variability in community maximum specific growth rate, yield, and mortality. Unlike time-based CV measures commonly used in stability assessments, we quantified the extent of variation among replicates under identical conditions. ANOVA was used to test the main and interactive effect of biodiversity (species richness, Faith's PD, and mean pairwise distance) and treatment on community growth, biomass, and mortality. To further elucidate these effects, we used the Student's t test to examine the effect of each environmental stressor on community growth, maximum yield, and death rates across the diversity gradient. We then fitted both linear and quadratic models to the BEF relationships, using the mean values of the ecosystem functioning metrics from the replicates for each combination. The Akaike Information Criterion (AIC) was employed for model comparison. Quadratic models with a ΔAIC (AIC_linear_ − AIC_quadratic_) >2 were considered significantly better, otherwise linear models were preferred. As biodiversity is usually in collinearity with the number of tolerant species in a given microcosm due to sampling effects, namely, microcosms that include many species have a higher probability of containing tolerant species, we used a linear mixed model with biodiversity as a random intercept. This approach allows us to account for the baseline differences in community structure arising from varying biodiversity levels and identify the effect of the absence of tolerant species on community growth, yield, and mortality. To infer species interactions, we compared observed community growth rates, yields, and mortality rates *A_j_* to the sum of those measured in monoculture of constituent species, ∑*a_i_w_i,j_*, where *a_i_* is the monoculture value of species *i* and *w_i,j_* indicates the relative contribution of species *i* in community *j* calculated according to the proportion of species *i* in the community *j* of the inoculum. A value of the ratio *A_j_*/∑*a_i_w_i,j_* of 1 indicates that ecosystem functioning traits are additive and hence there is no interaction. A value <1 indicates a negative interaction for growth rate and yield, and a positive interaction for mortality rate. Conversely, a value >1 indicates a positive interaction for growth rate and yield, and a negative interaction for mortality rate. For post hoc differentiation between control and treatment groups, Duncan's new multiple range test was applied.

## Supplementary Material

pgaf114_Supplementary_Data

## Data Availability

All raw datasets generated and analyzed during the course of this study and the codes for data analyses have been made publicly available at https://github.com/jiesi-lei/Diversity-producticity.
