# Supplementary material for: Revisiting the classical biodiversity–ecosystem functioning and stability relationships in microbial microcosms
Source: PNAS Nexus. 2025 Apr 5;4(4):pgaf114. doi: 10.1093/pnasnexus/pgaf114 (PMC12038814; doi:10.1093/pnasnexus/pgaf114)
Supplement: pgaf114_Supplementary_Data [file pgaf114_supplementary_data.pdf]

PNAS nexus

Supplementary Information for

Revisiting the classical biodiversity-ecosystem functioning and stability relationships in microbial microcosms

Jiesi Lei[1], Jiajie Feng[1,2], Junjun Ding[1,3], and Yunfeng Yang[1,4]*

[1]State Key Joint Laboratory of Environment Simulation and Pollution Control, School of Environment, Tsinghua University, Beijing 100084, China

[2]School of Biological Science and Medical Engineering, Beihang University, Beijing, 100191, China

[3]Key Laboratory of Dryland Agriculture, Ministry of Agriculture, Institute of Environment and Sustainable Development in Agriculture, Chinese Academy of Agricultural Sciences, Beijing 100081, China

[4]Institute of Environment and Ecology, Tsinghua Shenzhen International Graduate School, Tsinghua University, Shenzhen 518055, China

*To whom correspondence may be addressed. E-mail: yangyf@tsinghua.edu.cn; Phone: +86-010-62784692; Fax: +86-010-62794006

This PDF file includes:

Figures S1 to S6
Tables S1 to S2

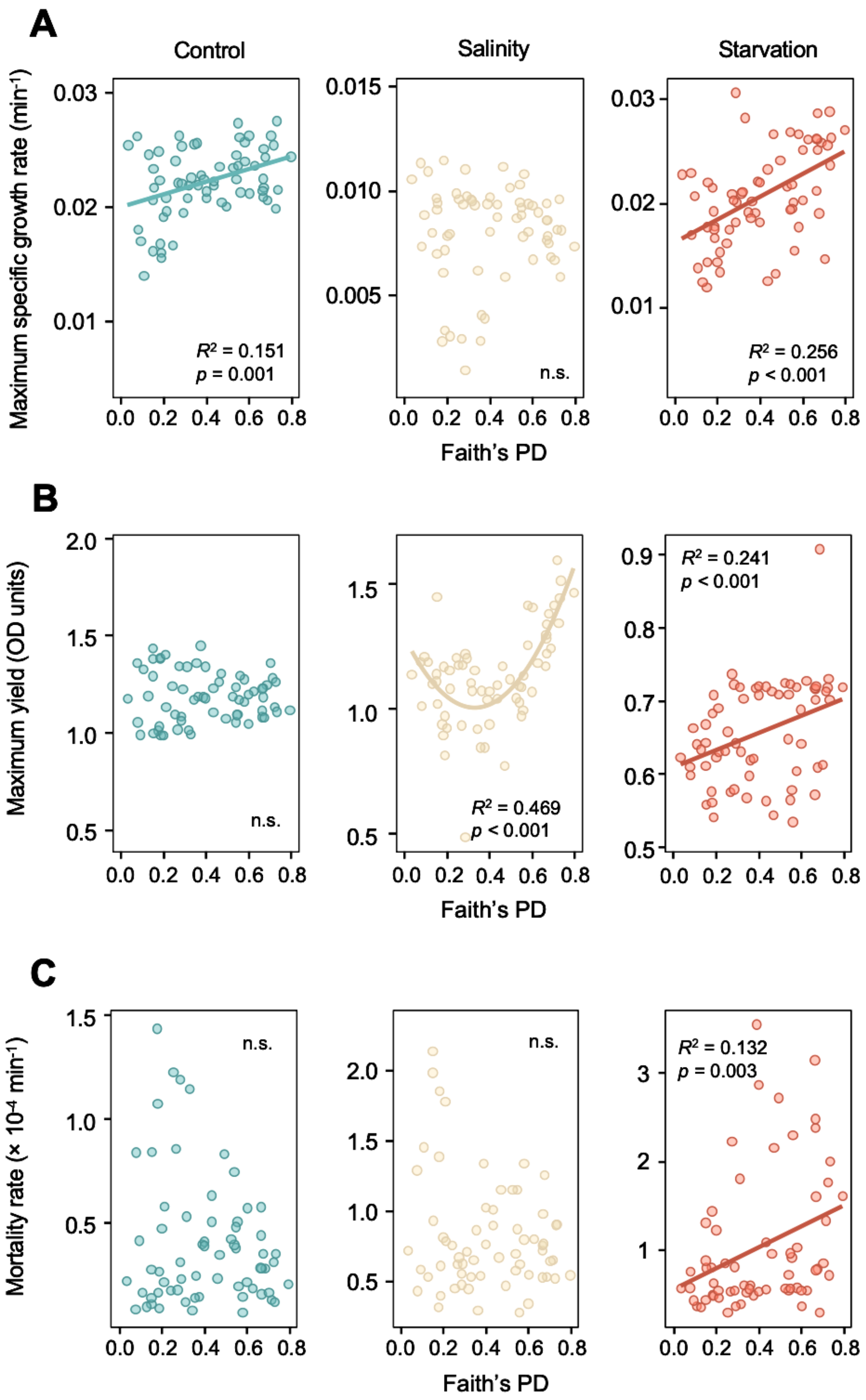

Fig. S1. Relationship between phylogenetic diversity and ecosystem functioning under different treatments. Ecosystem functioning includes (A) maximum specific growth rate, (B) maximum yield (OD_{600} values), and (C) mortality rates. Phylogenetic diversity is measured as Faith's PD. n.s. indicates that the regression is not significant at the 0.05 threshold, and the regression line is omitted.

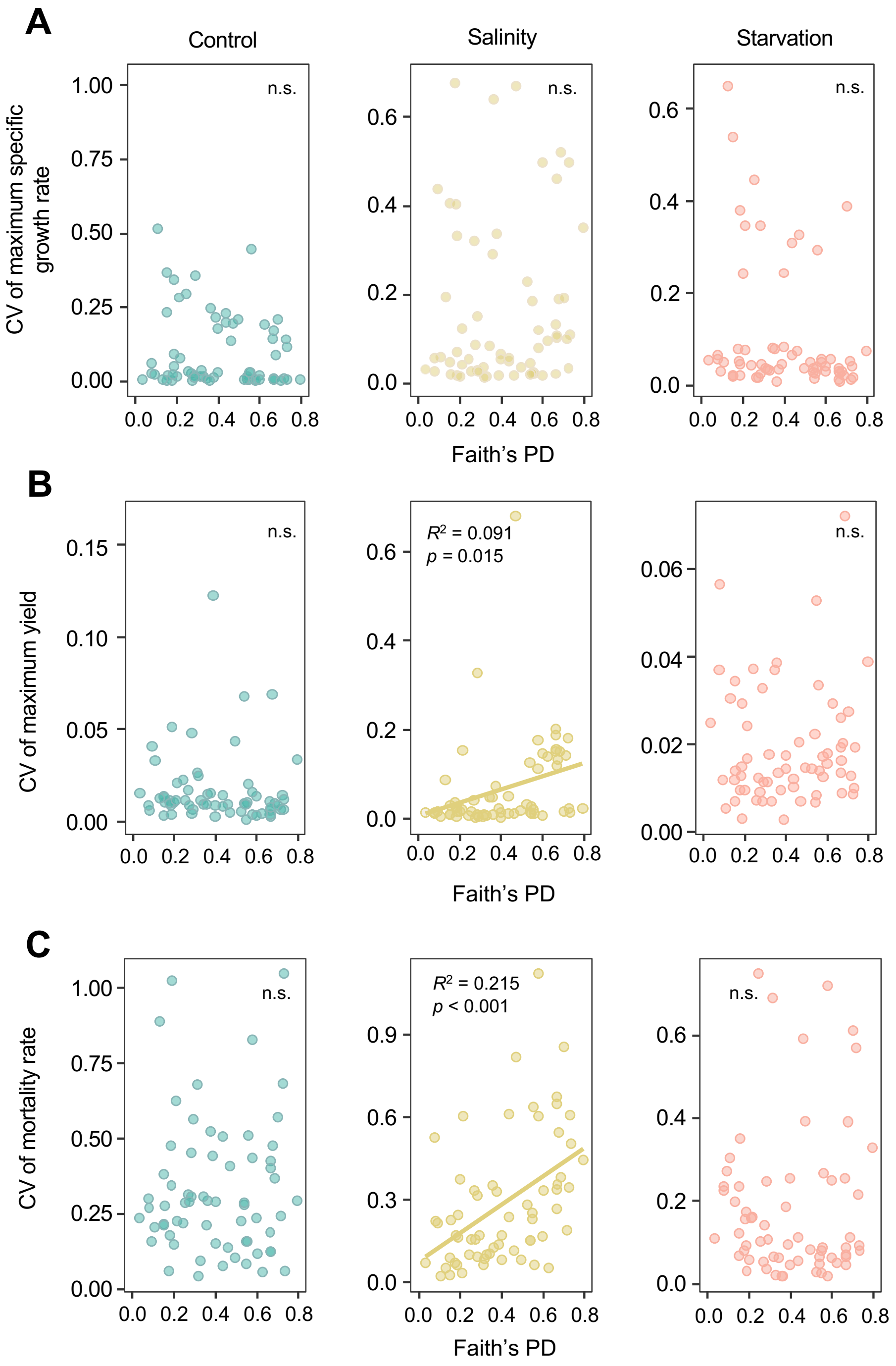

Fig. S2. Relationship between phylogenetic diversity and the CV of ecosystem functioning under different treatments. Ecosystem functioning includes (A) maximum specific growth rate, (B) maximum yield (OD_{600} values), and (C) mortality rates. Phylogenetic diversity is measured as Faith's PD. n.s. indicates that the regression is not significant at the 0.05 threshold, and the regression line is omitted.

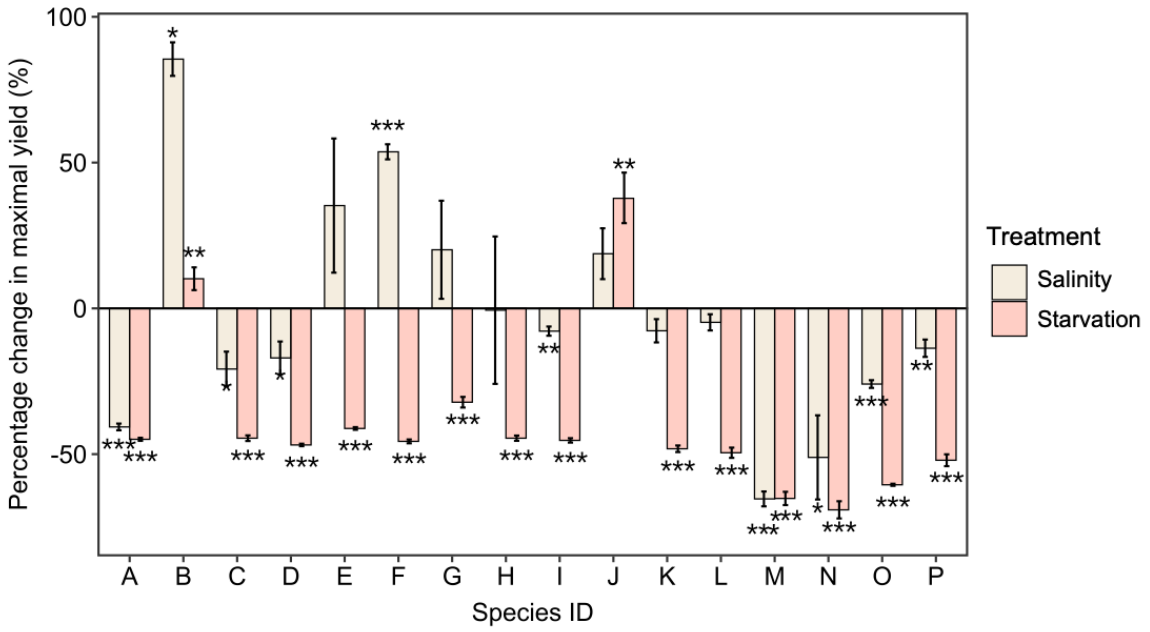

Fig. S3. Effects of treatments on maximum biomass (OD_{600}) of different species in monoculture. Negative bars indicate a percentage reduction and positive bars indicate a percentage increase compared to the control. A-P, species identities as shown in Supplementary Table 1. Error bars denote standard deviation. Bars followed by asterisks denote significant differences from the control: *** $p < 0.001$, ** $p < 0.010$, * $p < 0.050$.

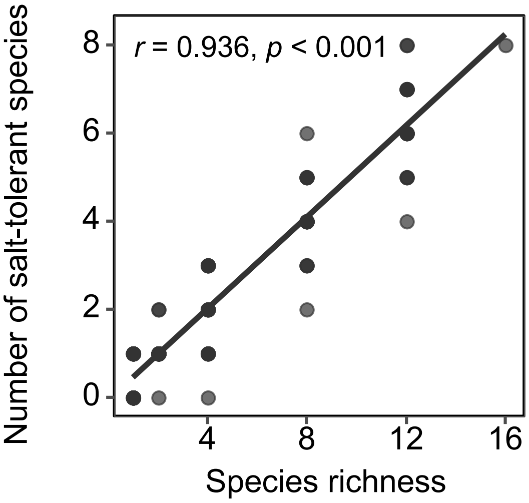

Fig. S4. Correlation between the number of salt-tolerant species and species richness.

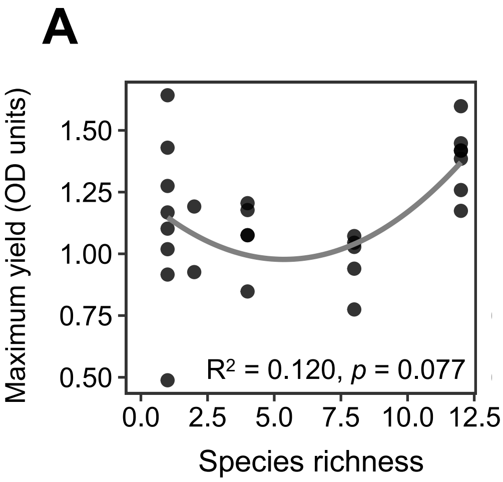

Fig. S5. Effects of biodiversity on maximum yield (OD_{600}) for communities dominated by salinity-tolerant species under saline stress. Biodiversity is measured as species richness in (A) and Faith's PD (phylogenetic diversity) in (B).

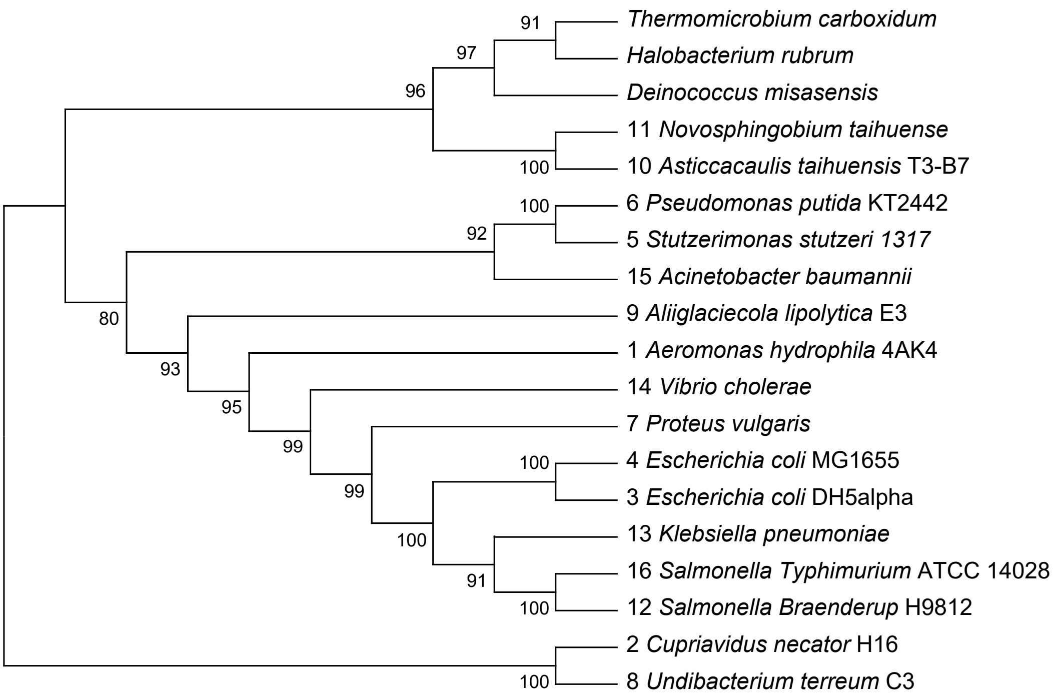

Fig. S6. Maximum likelihood phylogenetic tree (50% majority-rule consensus tree) showing the relationship among 16 bacterial species based on 16S rRNA gene sequences. Numbers at nodes indicate the bootstrap values.

Table S1. Bacterial species used in the study.

No.	Species ID	Strain	Class	Estimated *rrn* copy number
1	A	*Aeromonas hydrophila* 4AK4	γ-Proteobacteria	9
2	B	*Cupriavidus necator* H16	β-Proteobacteria	3
3	C	*Escherichia coli* DH5α	γ-Proteobacteria	4
4	D	*Escherichia coli* MG1655	γ-Proteobacteria	7
5	E	*Stutzerimonas stutzeri* 1317	γ-Proteobacteria	4
6	F	*Pseudomonas putida* KT2442	γ-Proteobacteria	7
7	G	*Proteus vulgaris*	γ-Proteobacteria	7
8	H	*Undibacterium terreum* C3	β-Proteobacteria	4
9	I	*Aliiglaciecola lipolytica* E3	γ-Proteobacteria	4
10	J	*Asticcacaulis taihuensis* T3-B7	α-Proteobacteria	3
11	K	*Novosphingobium taihuense*	α-Proteobacteria	3
12	L	*Salmonella Braenderup* H9812	γ-Proteobacteria	7
13	M	*Klebsiella pneumoniae*	γ-Proteobacteria	8
14	N	*Vibrio cholerae*	γ-Proteobacteria	9
15	O	*Acinetobacter baumannii*	γ-Proteobacteria	6
16	P	*Salmonella Typhimurium* ATCC 14028	γ-Proteobacteria	7

Table S2. Species composition and diversity of artificial communities.

Composition	Richness	Phylogenetic diversity	Mean pairwise distance	A	B	C	D	E	F	G	H	I	J	K	L	M	N	O	P
1	1	n.a.*	n.a.	1	0	0	0	0	0	0	0	0	0	0	0	0	0	0	0
2	1	n.a.	n.a.	0	1	0	0	0	0	0	0	0	0	0	0	0	0	0	0
3	1	n.a.	n.a.	0	0	1	0	0	0	0	0	0	0	0	0	0	0	0	0
4	1	n.a.	n.a.	0	0	0	1	0	0	0	0	0	0	0	0	0	0	0	0
5	1	n.a.	n.a.	0	0	0	0	1	0	0	0	0	0	0	0	0	0	0	0
6	1	n.a.	n.a.	0	0	0	0	0	1	0	0	0	0	0	0	0	0	0	0
7	1	n.a.	n.a.	0	0	0	0	0	0	1	0	0	0	0	0	0	0	0	0
8	1	n.a.	n.a.	0	0	0	0	0	0	0	1	0	0	0	0	0	0	0	0
9	1	n.a.	n.a.	0	0	0	0	0	0	0	0	1	0	0	0	0	0	0	0
10	1	n.a.	n.a.	0	0	0	0	0	0	0	0	0	1	0	0	0	0	0	0
11	1	n.a.	n.a.	0	0	0	0	0	0	0	0	0	0	1	0	0	0	0	0
12	1	n.a.	n.a.	0	0	0	0	0	0	0	0	0	0	0	1	0	0	0	0
13	1	n.a.	n.a.	0	0	0	0	0	0	0	0	0	0	0	0	1	0	0	0
14	1	n.a.	n.a.	0	0	0	0	0	0	0	0	0	0	0	0	0	1	0	0
15	1	n.a.	n.a.	0	0	0	0	0	0	0	0	0	0	0	0	0	0	1	0
16	1	n.a.	n.a.	0	0	0	0	0	0	0	0	0	0	0	0	0	0	0	1
17	2	0.174	0.174	1	0	0	0	0	0	0	1	0	0	0	0	0	0	0	0
18	2	0.105	0.105	1	0	0	0	0	0	0	0	0	0	0	0	0	0	0	1

19	2	0.185	0.185	0	1	1	0	0	0	0	0	0	0	0	0	0	0	0	0
20	2	0.181	0.181	0	1	0	0	0	0	0	0	0	0	0	0	0	0	0	1
21	2	0.151	0.151	0	0	1	0	0	1	0	0	0	0	0	0	0	0	0	0
22	2	0.076	0.076	0	0	1	0	0	0	1	0	0	0	0	0	0	0	0	0
23	2	0.030	0.030	0	0	0	1	0	0	0	0	0	0	0	1	0	0	0	0
24	2	0.197	0.197	0	0	0	0	1	0	0	0	0	1	0	0	0	0	0	0
25	2	0.148	0.148	0	0	0	0	1	0	0	0	0	0	0	0	0	0	0	1
26	2	0.148	0.148	0	0	0	0	0	1	0	0	0	0	0	0	0	1	0	0
27	2	0.147	0.147	0	0	0	0	0	1	0	0	0	0	0	0	0	0	0	1
28	2	0.073	0.073	0	0	0	0	0	0	1	0	0	0	0	1	0	0	0	0
29	2	0.178	0.178	0	0	0	0	0	0	0	1	1	0	0	0	0	0	0	0
30	2	0.184	0.184	0	0	0	0	0	0	0	1	0	0	0	0	0	0	1	0
31	2	0.210	0.210	0	0	0	0	0	0	0	0	0	1	0	0	1	0	0	0
32	2	0.207	0.207	0	0	0	0	0	0	0	0	0	1	0	0	0	0	0	1
33	4	0.264	0.134	1	0	0	0	0	1	0	0	1	0	0	0	1	0	0	0
34	4	0.359	0.184	1	0	0	0	0	0	1	1	0	1	0	0	0	0	0	0
35	4	0.282	0.152	0	1	1	0	0	0	0	0	0	0	0	0	0	0	1	1
36	4	0.251	0.139	0	1	0	0	0	1	0	0	0	0	0	1	0	0	0	1
37	4	0.326	0.166	0	0	0	1	1	0	0	0	0	0	1	0	0	0	1	0
38	4	0.089	0.047	0	0	0	1	0	0	1	0	0	0	0	1	0	0	0	1
39	4	0.308	0.160	0	0	0	1	0	0	0	1	0	0	0	0	0	1	1	0

40	4	0.240	0.125	0	0	0	1	0	0	0	1	0	0	0	0	0	1	0	1
41	4	0.315	0.159	0	0	0	0	0	1	0	1	1	0	0	1	0	0	0	0
42	4	0.288	0.156	0	0	0	0	0	1	0	0	0	1	0	1	1	0	0	0
43	4	0.372	0.192	0	0	0	0	0	0	0	1	0	1	1	0	1	0	0	0
44	4	0.271	0.140	0	0	0	0	0	0	0	0	1	0	0	0	0	1	1	1
45	4	0.340	0.175	1	1	1	0	0	0	0	0	0	0	1	0	0	0	0	0
46	4	0.127	0.065	1	0	1	1	0	0	0	0	0	0	0	1	0	0	0	0
47	4	0.282	0.145	1	0	0	1	0	0	1	0	0	0	1	0	0	0	0	0
48	4	0.353	0.181	1	0	0	1	0	0	0	1	0	1	0	0	0	0	0	0
49	8	0.553	0.159	1	1	0	0	1	0	0	0	1	1	0	1	0	0	1	1
50	8	0.596	0.168	1	1	0	0	1	0	0	1	1	1	0	0	0	0	1	1
51	8	0.458	0.138	1	1	1	0	0	1	0	0	1	0	0	0	1	0	1	1
52	8	0.557	0.160	1	1	1	1	0	1	0	0	1	1	0	0	0	0	1	0
53	8	0.491	0.144	0	0	0	1	0	1	0	1	1	0	1	1	1	1	0	0
54	8	0.545	0.156	0	0	0	1	1	0	0	1	1	0	1	1	0	1	1	0
55	8	0.468	0.147	0	0	1	0	0	1	1	0	1	1	1	1	0	0	0	1
56	8	0.537	0.157	0	0	1	0	1	0	1	1	0	1	0	0	0	1	1	1
57	8	0.385	0.131	0	0	1	1	1	1	1	1	0	0	0	0	0	0	1	1
58	8	0.536	0.165	0	1	0	0	1	1	1	0	0	1	1	0	1	1	0	0
59	8	0.396	0.130	0	1	0	1	1	0	1	1	0	0	0	1	0	1	0	1
60	8	0.432	0.128	0	1	1	0	0	0	1	0	1	0	0	1	0	1	1	1

61	8	0.396	0.130	0	1	0	1	1	0	1	1	0	0	0	1	0	1	0	1
62	8	0.432	0.128	0	1	1	0	0	0	1	0	1	0	0	1	0	1	1	1
63	8	0.522	0.165	0	1	1	1	1	1	0	0	0	1	1	0	0	0	1	0
64	8	0.544	0.162	1	0	1	0	0	0	1	1	1	1	1	0	1	0	0	0
65	12	0.597	0.142	1	0	1	1	1	1	0	1	1	1	1	1	1	0	0	1
66	12	0.683	0.145	1	0	1	1	0	0	1	1	1	1	1	0	1	1	1	1
67	12	0.721	0.152	1	0	1	0	1	0	1	1	1	1	1	1	0	1	1	1
68	12	0.665	0.139	1	0	1	0	0	1	1	1	1	1	0	1	1	1	1	1
69	12	0.730	0.156	1	0	0	1	1	1	1	1	1	1	1	0	1	1	1	0
70	12	0.663	0.139	1	0	0	1	1	0	1	1	1	1	0	1	1	1	1	1
71	12	0.662	0.151	0	1	1	1	1	1	1	1	1	1	0	0	0	1	1	1
72	12	0.661	0.155	0	1	0	1	1	1	1	1	1	1	1	1	0	1	0	1
73	12	0.673	0.156	1	1	1	1	1	1	1	1	1	1	1	0	1	0	0	0
74	12	0.622	0.146	1	1	1	1	0	0	1	1	0	1	1	1	1	0	1	1
75	12	0.576	0.137	1	1	1	0	1	1	1	1	0	0	1	1	1	1	0	1
76	12	0.712	0.160	1	1	1	0	1	1	1	1	0	1	1	1	0	1	1	0
77	12	0.576	0.137	1	1	1	0	1	1	1	1	0	0	1	1	1	1	0	1
78	12	0.666	0.139	1	1	1	0	1	0	1	0	1	1	0	1	1	1	1	1
79	12	0.700	0.154	1	1	0	1	1	0	1	1	1	1	1	1	0	1	0	1
80	12	0.726	0.156	1	1	0	0	1	1	1	0	1	1	1	1	1	1	1	0
81	16	0.792	0.146	1	1	1	1	1	1	1	1	1	1	1	1	1	1	1	1

*n.a., not applicable
